# Acupuncture for the treatment of leptin resistance in obesity: A protocol for systematic review and meta-analysis

**DOI:** 10.1097/MD.0000000000026244

**Published:** 2021-07-16

**Authors:** Tianjiao Gao, Dehui Ma, Shaotao Chen, Xiaolin Zhang, Yiran Han, Mingjun Liu

**Affiliations:** a Changchun University of Chinese Medicine; b Acupuncture and Massage Center of the Third Affiliated Clinical Hospital of Changchun University of Chinese Medicine, Changchun, China.

**Keywords:** acupuncture, leptin resistance, meta-analysis, obesity, systematic review

## Abstract

**Background:**

Recently, there has been a global increase in obesity and obesity-related diseases. The prevention and treatment of obesity have become one of the most significant public health challenges worldwide in the 21st century, and how to effectively curb the occurrence of obesity has become a major global concern. Numerous studies have shown that the majority of obese individuals do not respond to leptin, and instead demonstrate leptin resistance. Clinical studies have found that acupuncture is widely used in the clinical treatment of obesity in recent years, but whether it can improve leptin resistance has not been systematically reviewed. This study is aimed to investigate the effectiveness of acupuncture in obesity with leptin resistance (LR).

**Methods:**

We searched PubMed, Cochrane Library, Embase, China National Knowledge Infrastructure (CNKI), Wan Fang, Technology Journal Database (VIP), and China Biology Medicine disc (CBM). Chinese Clinical Trial Registry. The time was from the establishment of the database to March 19, 2021. RevMan 5.3 software was used to assess the quality and risk of the included studies.

**Results:**

This study will be conducted in terms of clinical efficacy, serum leptin content, and body weight change. The current evidence shows that the incidence of the disease is high and the comprehensive quality is high.

**Conclusion:**

The conclusion of this review will provide a basis for judging whether acupuncture therapy is effective in the treatment of leptin resistance in obesity.

## 1 Introduction

Obesity is a dysmetabolic disease in which body weight exceeds the normal range as a result of long-term energy intake greater than body expenditure and excessive body fat accumulation, in addition to being a risk factor for chronic diseases such as diabetes, hypertension, coronary heart disease, and stroke,^[1]^ and recent years have witnessed a global increase in obesity and obesity-related diseases. The prevention and treatment of obesity have become one of the most significant public health challenges worldwide in the 21st century, and how to effectively curb the occurrence of obesity has become a major global concern^[2]^ and elucidating the mechanisms underlying obesity is critical for understanding the occurrence and progression of obesity.^[3]^

Leptin levels are increased in the circulation of obese individuals, but failed to exert appetite suppression, food lowering, and thermogenic effects in vivo, leading some to propose that leptin resistance also occurs in obesity. Leptin resistance (LR), defined by the reduced ability of leptin to suppress appetite and weight gain, is often observed in obese individuals, and serum levels of leptin decrease with reductions in body weight.^[4]^

Traditional Chinese medicine (TCM) internal medicine and acupuncture are effective in the treatment of obesity.^[5]^ Acupuncture has been hailed as a green therapy, and many clinical studies and experimental studies have shown that TCM acupuncture, as a healthy, effective, and safe treatment measure, has a good treatment effect on obesity, but the mechanism of acupuncture's weight loss effect has not been clearly reported so far. Some studies have shown that leptin serum levels can be obtained by acupuncture in obese people. However, there is no systematic review of acupuncture for the treatment of leptin resistance in obesity, and this is the first randomized controlled trial using meta-analysis to explore the effects of acupuncture interventions in obese leptin resistant patients.^[6]^

## 2 Data and methods

The study has been registered in INPLASY with the registration number of INPLASY202150056.

### 2.1 Search strategy

PubMed, Cochrane Library, Embase databases, China National Knowledge Infrastructure(CNKI), WanFang, Technology Journal Database(VIP), China Biology Medicine disc(CBM), and Chinese Clinical Trial Registry (ChiCTR) were searched with no language restrictions. By using the combination of subject words and free words. The time was from the establishment of the database to March 19, 2021.English search words are “obesity,” “overweight, ” “Leptin resistance, ” “acupuncture, ” “Acupuncture Treatment,” “randomized controlled trial,” “controlled clinical trial,” “randomized” and so on; Chinese search words are “obesity,” “leptin resistance,” “acupuncture,” “ randomized controlled trial,” “controlled clinical trial” and so on. We also manually searched the references of relevant studies to further identify relevant literature. Table 1 shows the search strategy in PubMed database.

**Table 1:** Search strategy used in Pubmed database.

Search strategy used in Pubmed database.	
Number	Search terms
1	Obesity. Mesh
2	Overweight. Ti, ab
3	Adiposity. Ti, ab
4	adiposis. Ti, ab
5	nutrition disorders. Ti, ab
6	Overnutrition. Ti, ab
7	Simple obesity. Ti, ab
8	Obesity obese. Ti, ab
9	Nutritional and Metabolic Diseases. Ti, ab
10	Weight loss. Ti, ab
11	Weight control. Ti, ab
12	OR 1–11
13	Leptin resistance. Ti, ab
14	Acupuncture. Mesh
15	manual acupuncture. Ti, ab
16	electro-acupuncture. Ti, ab
17	auricular acupuncture. Ti, ab
18	acupoint injection. Ti, ab
19	Pharmacopuncture. Ti, ab
20	Acupuncture Treatment. Ti, ab
21	Acupuncture Treatments. Ti, ab
22	Acupotomy. Ti, ab
23	Acupotomies. Ti, ab
24	OR 14–23
25	randomized controlled trial. Pt
26	controlled clinical trial. Pt
27	Randomized. Ti, ab
28	Randomly. Ti, ab
29	Placebo. Ti, ab
30	OR 25–29
31	12 and 13 and 24 and 30

The search strategy will be modified as required for other electronic database.

### 2.2 Inclusion criteria

#### 2.2.1 Types of studies

The type of literature gathered must be based on RCT trials. Do not limit the use of blind methods, but the authors should clearly claim that they performed random grouping.

#### 2.2.2 Types of participants

According to the diagnostic criteria, all participants were diagnosed with obesity with LR. The age, sex, course, number, and ethnicity of the participants were not limited.

#### 2.2.3 Types of interventions

Patients in the experimental groups received needle acupuncture as the main intervention which included manual acupuncture, electro-acupuncture, auricular acupuncture and acupoint injection. The control group was treated with the oral medicine, placebo, sham acupuncture, or lifestyle modification.

#### 2.2.4 Outcome indicators

At least including serum leptin level, BW, body mass index (BMI), waist-to-hip ratio (WHR), and any two items of effective rate (effective rate = (recovery + significant effect + effective)/total number of cases].

### 2.3 Study screening and data extraction

**Figure 1:**
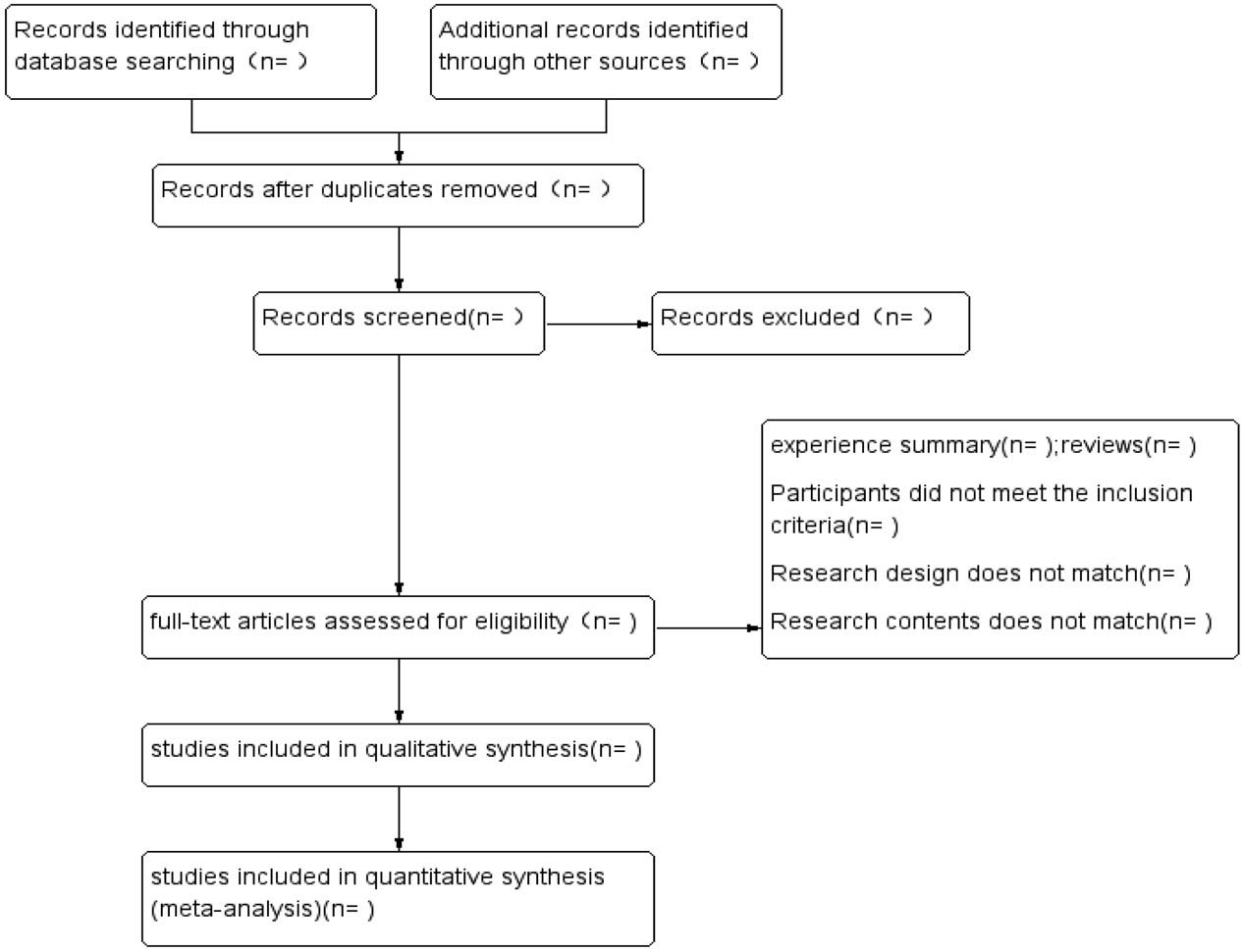
PRISMA flow diagram of study and exclusion.

All studies retrieved from the databases were entered into Endnote. Duplicates were identified and deleted. Then, two reviewers (Gao Tianjiao and Ma Dehui) independently screened the titles and abstract of the database records. A data extraction table was constructed which included the author, the years, the country or region, the age, number of patients, samples, interventions, treatment time, outcome indicators and information related to bias risk assessment. These data were extracted by two reviewers independently. Differences were resolved by discussing with Chen Shaotao, the third reviewer. The literature screening process was illustrated in Figure 1.

### 2.4 Statistical analysis

We will use Review Managerversion5.3, to create a flow chart and PRISMA scale.^[7]^ Revman 5.3 and Stata 16 software were used for the meta-analysis. Weighted mean differences (WMDs) were calculated with 95% confidence intervals (CIs) for continuous variables. The Q test and I^2^ test were used to assess the heterogeneity of the data. When heterogeneity was not significant (*P* ≥ .10 or I^2^ < 50%), a fixed effects model was used to analyze the pooled effect, and when heterogeneity was statistically significant (I^2^ ≥50% or *P* < .10), a random effects model was used^[8]^ Reasons for heterogeneity were explored by subgroup analysis. Forest plots were generated to illustrate the corresponding changes in treatment effects.

#### 2.4.1 Risk assessment of literature bias

The risk of bias of literature was adopted by two reviewers (Gao Tianjiao and Ma Dehui) using the Cochrane tool.^[9]^ Each study was scored as high, low, or unclear risk of seven items:

1. generation of a random allocation scheme;
2. allocation scheme concealment;
3. blind method for participants and doctors;
4. blind method for outcome evaluation;
5. incomplete outcome assessment;
6. selective reporting of study results; and
7. other sources of bias; if more than three of these items were rated as low risk, the quality of the entire trial was classified as low risk, and the quality of the entire trial was classified as low risk if more than three of these items were rated as low-risk. If more than three items were identified as high or unclear risk, the overall quality was classified as high risk. A third reviewer was consulted in case of disagreement.

#### 2.4.2 Assessment of publication bias

If the meta-analysis results contained more than 10 articles, the funnel plot was drawn and analyzed using revman 5.3, and publication bias was checked by plotting the funnel map. Funnel plot symmetry means that there is no publication bias, and asymmetry is the opposite.

#### 2.4.3 Quality of evidence

The primary outcomes will be assessed by the grading of recommendations in assessment as well as the development of assessment methodology. Assessments included the risk of bias, heterogeneity, indirectness, imprecision, and publication bias.

## 3 Discussion

Therapeutic tracking of obesity with oral agents, especially hormonal agents, has certain advantages. Western medicine often encourages the gastrointestinal tract, and long-term administration of drugs can produce adverse effects. The search for an effective and safe treatment and improvement of the prognosis of leptin resistance in obese patients is an important health issue. Acupuncture, as an alternative medicine, can reduce fat synthesis and improve physiological function to normal people in obese patients with leptin resistance. The relevant experimental results of traditional Chinese medicine acupuncture showed that after acupuncture treatment, serum leptin decreased significantly and could effectively improve the leptin resistance state in obese subjects.^[10]^

As there is currently no systematic meta-analysis of acupuncture in the treatment of leptin resistance in obesity, it is hoped that these results may provide clinicians with a basis for acupuncture in the treatment of leptin resistance in obesity and provide support for the effectiveness of acupuncture in the treatment of leptin resistance in obesity. There may be incomplete literature due to the inability to search all databases and unpublished studies; moreover, this study only included Chinese and English literature, which may have caused selection bias. This study may provide a basis for relevant studies on the acupuncture treatment of leptin resistance in obesity.

## Author contributions

**Conceptualization:** Tianjiao Gao, Mingjun Liu.

**Data curation:** Shaotao Chen, Xiaolin Zhang, Yiran Han.

**Formal analysis:** Shaotao Chen.

**Methodology:** Dehui Ma, Shaotao Chen.

**Software:** Tianjiao Gao, Yiran Han.

**Supervision:** Mingjun Liu.

**Writing – original draft:** Dehui Ma, Xiaolin Zhang, Mingjun Liu.

**Writing – review & editing:** Tianjiao Gao.
